# Supplementary material for: Quantum spin Hall phase in 2D trigonal lattice
Source: Nat Commun. 2016 Sep 7;7:12746. doi: 10.1038/ncomms12746 (PMC5023954; doi:10.1038/ncomms12746)
Supplement: Supplementary Information — Supplementary Figure 1-2 and Supplementary Note 1-2 [file ncomms12746-s1.pdf]

Supplementary Figure 1

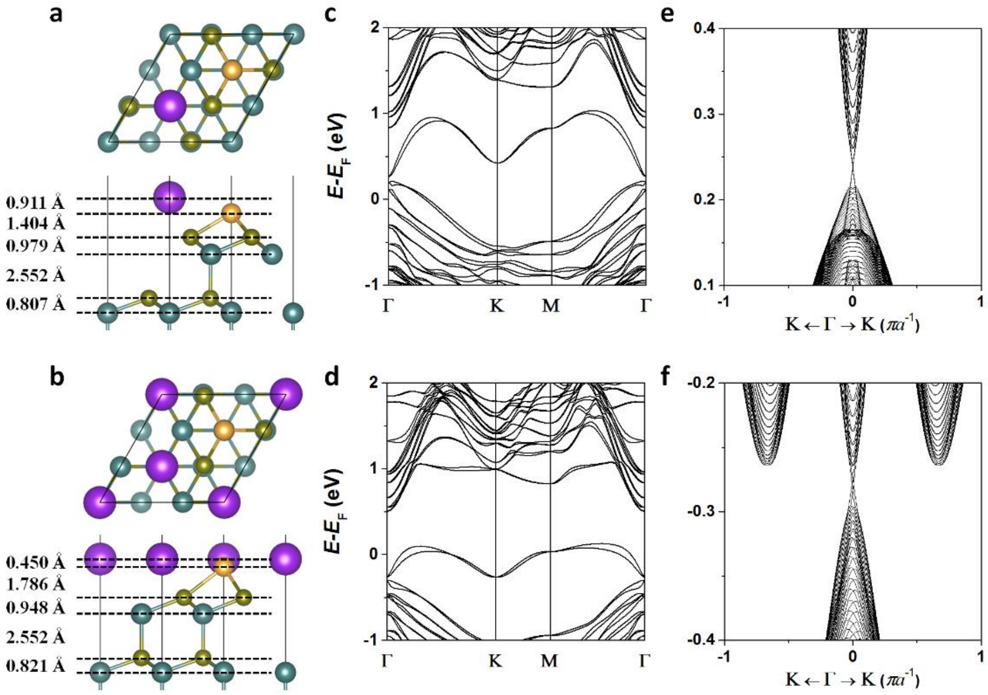

Supplementary Figure 1 | Au/GaAs(111) with surface n-doping. a,b, Top and side view of atomic structure of Au/GaAs(111) at 1/3 and 2/3 coverage of K atoms. The labels denote the layer distance between different atoms. **c,d,** Band structure of a and b with SOC. **e,f,** 1D ribbon band structure of a and b, showing the gapless Dirac edge states within the energy window of SOC gap.

Supplementary Figure 2

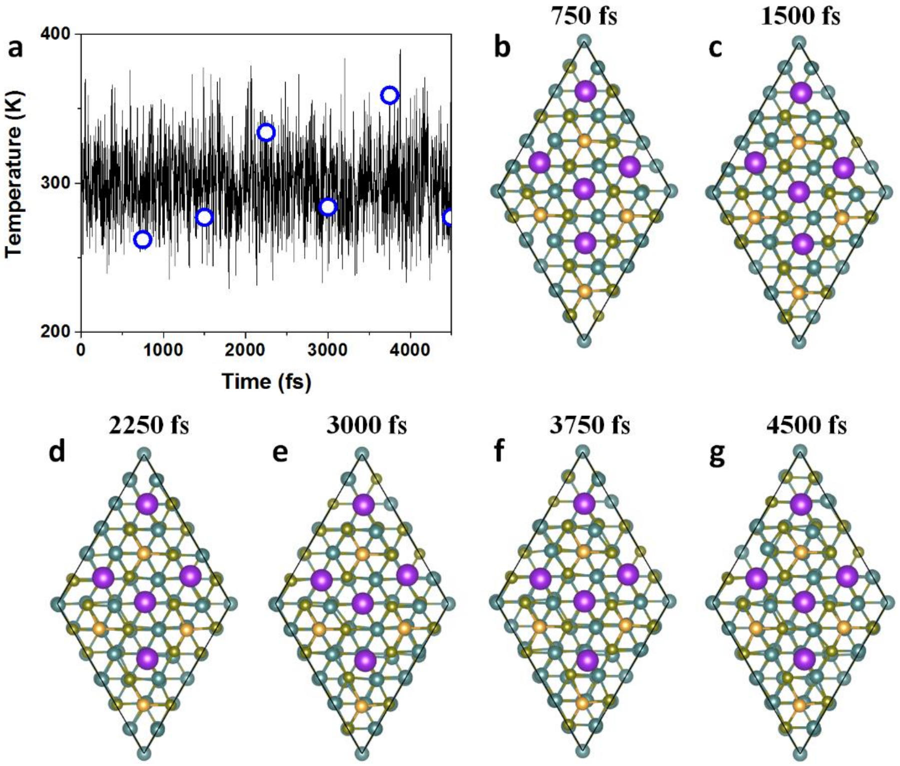

Supplementary Figure 2 | Stability of K atoms on Au/GaAs(111) surface. a, Temperature variation with time, and the open blue dots denote the times of snap shots taken in b-g. The first-principles Molecular dynamics (MD) simulations for Au/GaAs(111) at 5/12 coverage of K atoms are done using canonical ensemble at 300 K. **b-g**, Snap shots of the structures at different times, demonstrating the stability of surface K atoms at room temperature.

Supplementary Note 1

To design a minimal basis QSH model, we have to determine two factors. One is the lattice symmetry and the other is the atomic orbitals. Generally, at least two orbitals with different symmetry (odd or even) are needed for realizing a two-band inversion at the continuum limit. Consequently, in the classical Kane-Mele model, the minimal basis consists of one p_z orbital, because there are two lattice sites per unit cell in a bipartite hexagonal lattice, to produce two bands of opposite parity (π, π*). For a trigonal lattice having one lattice site per unit-cell, clearly one orbital per lattice site could not produce a non-trivial band topology because there would be only one band. Thus, at least two orbitals of different symmetry are requires. Take the simplest case of *s* and *p* orbitals, one can set one *s* and one *p* orbital per lattice site. Because *s* and p_z orbitals are decoupled from each other in the planar geometry, one must choose one *s* and one p_x/p_y orbital. However, the energy of p_x and p_y orbitals are degenerated without breaking the in-plane symmetry. Therefore, as a generic model, the minimal basis for QSH phase in a trigonal lattice consists of three orbitals (*s*, p_x, p_y).

In the basis of (*s*, p_x, p_y), the corresponding spinless TB Hamiltonian for the trigonal lattice can be written as

$$H_0 = \sum_{\alpha} \varepsilon_{\alpha} c_{0\alpha}^{+} c_{0\alpha} + \sum_{i} \sum_{\alpha,\beta} (t_{0\alpha,i\beta} c_{0\alpha}^{+} c_{i\beta} + t_{i\beta,0\alpha} c_{i\beta}^{+} c_{0\alpha}) , \quad (1)$$

where $\alpha,\beta=s$, p_x, p_y are the orbital index, ε_α is the on-site energy and $t_{0\alpha,i\beta}$ is the NN hopping parameter. c^+ and c are creation and annihilation operators. The on-site SOC term can be written as

$$H_{\mathrm{SOC}} = -i\lambda(c^+_{0p_x} c_{0p_y} - c^+_{0p_y} c_{0p_x}) \cdot s_z \, , \tag{2}$$

where λ is the SOC strength, s_z is the Pauli matrix and i is the imaginary unit. Since spin-up and spin-down Hamiltonians are decoupled, without losing the generality, we will focus on the spin-up part (one just needs to change λ to $-\lambda$ for the spin-down Hamiltonian). The spin-up Hamiltonian can be written as

$$H = H_0 + H_{\mathrm{soc}} = \begin{pmatrix} H_{11} & H_{12} & H_{13} \\ H_{12}^* & H_{22} & H_{23} \\ H_{13}^* & H_{23}^* & H_{33} \end{pmatrix} + \begin{pmatrix} 0 & 0 & 0 \\ 0 & 0 & -i\lambda \\ 0 & i\lambda & 0 \end{pmatrix}, \tag{3}$$

where the matrix elements are

$$\begin{aligned}
H_{11} &= \varepsilon_s + t_{ss\sigma}[4\cos(\frac{\sqrt{3}}{2}k_x)\cos(\frac{1}{2}k_y) + 2\cos(k_y)] \\
H_{12} &= t_{sp\sigma}[2\sqrt{3}i\sin(\frac{\sqrt{3}}{2}k_x)\cos(\frac{1}{2}k_y)] \\
H_{13} &= t_{sp\sigma}[2i\cos(\frac{\sqrt{3}}{2}k_x)\sin(\frac{1}{2}k_y) + 2i\sin(k_y)] \\
H_{22} &= \varepsilon_p + (3t_{pp\sigma} + t_{pp\pi})\cos(\frac{\sqrt{3}}{2}k_x)\cos(\frac{1}{2}k_y) + 2t_{pp\pi}\cos(k_y) \\
H_{23} &= (t_{pp\pi} - t_{pp\sigma})\sqrt{3}\sin(\frac{\sqrt{3}}{2}k_x)\sin(\frac{1}{2}k_y) \\
H_{33} &= \varepsilon_p + (t_{pp\sigma} + 3t_{pp\pi})\cos(\frac{\sqrt{3}}{2}k_x)\cos(\frac{1}{2}k_y) + 2t_{pp\sigma}\cos(k_y)
\end{aligned} \, , \tag{4}$$

where ε_s and ε_p are the on site energy for s and p orbital, respectively. $t_{ss\sigma}$, $t_{sp\sigma}$, $t_{pp\sigma}$ and $t_{pp\pi}$ are NN hopping parameter, and λ is the SOC strength. One may also change the basis to (s, p_x+ip_y, p_x-ip_y), then the spin-up Hamiltonian becomes

$$H = \begin{pmatrix} H_{11} & \frac{1}{\sqrt{2}}(H_{12} + iH_{13}) & \frac{1}{\sqrt{2}}(H_{12} - iH_{13}) \\ \frac{1}{\sqrt{2}}(H_{12}^* - iH_{13}^*) & \frac{1}{2}(H_{22} + iH_{23} - iH_{23}^* + H_{33}) + \lambda & \frac{1}{2}(H_{22} - iH_{23} - iH_{23}^* - H_{33}) \\ \frac{1}{\sqrt{2}}(H_{12}^* + iH_{13}^*) & \frac{1}{2}(H_{22} + iH_{23} + iH_{23}^* - H_{33}) & \frac{1}{2}(H_{22} - iH_{23} + iH_{23}^* + H_{33}) - \lambda \end{pmatrix}, \quad (5)$$

Around the Γ point, the above Hamiltonian can be expended to the first-order of k as

$$H = \begin{pmatrix} \varepsilon_s + 6t_{ss\sigma} & \frac{3}{\sqrt{2}} t_{sp\sigma}(ik_x - k_y) & \frac{3}{\sqrt{2}} t_{sp\sigma}(ik_x + k_y) \\ \frac{3}{\sqrt{2}} t_{sp\sigma}(-ik_x - k_y) & \varepsilon_p + 3(t_{pp\sigma} + t_{pp\pi}) + \lambda & 0 \\ \frac{3}{\sqrt{2}} t_{sp\sigma}(-ik_x + k_y) & 0 & \varepsilon_p + 3(t_{pp\sigma} + t_{pp\pi}) - \lambda \end{pmatrix}, \quad (6)$$

Supplementary Note 2

Suppose we have two Hamiltonian H_{00} and H_{11}, and the interaction between them is H_{01} and H_{10}, its Green's function can be written as

$$\begin{pmatrix} E - H_{00} & -H_{01} \\ -H_{10} & E - H_{11} \end{pmatrix} \begin{pmatrix} G_{00} & G_{01} \\ G_{10} & G_{11} \end{pmatrix} = \begin{pmatrix} I & 0 \\ 0 & I \end{pmatrix}, \quad (7)$$

From the second column of the Green's function, we have the relation

$$\begin{aligned} (E - H_{00})G_{01} - H_{01}G_{11} = 0 \\ -H_{10}G_{01} + (E - H_{11})G_{11} = I \end{aligned}, \quad (8)$$

Therefore,

$$\left\{E - [H_{11} + H_{10}(E - H_{00})^{-1} H_{01}]\right\} G_{11} = I, \quad (9)$$

So the effective Hamiltonian of H_{11} can be written as

$$H_{11}^{\mathrm{eff}} = H_{11} + H_{10}(E - H_{00})^{-1} H_{01}, \quad (10)$$

where the second term is the effective contribution of H_{00} to H_{11}, and E is the chemical potential. Using the relation of Supplementary Equation (10), we can reduce our three-band minimal basis Hamiltonian to an effective two-band Hamiltonian.

Starting from Supplementary Equation (5), if we eliminate the bottom (p_x–ip_y) orbital for the first type band ($E_s > E_p$), the effective two-band Hamiltonian can be written as

$$H_{\text{eff}} = \begin{pmatrix} H_{11} & \frac{1}{\sqrt{2}}(H_{12}+iH_{13}) \\ \frac{1}{\sqrt{2}}(H_{12}^{*}-iH_{13}^{*}) & \frac{1}{2}(H_{22}+iH_{23}-iH_{23}^{*}+H_{33})+\lambda \end{pmatrix} + \frac{\begin{pmatrix} \frac{1}{\sqrt{2}}(H_{12}-iH_{13}) \\ \frac{1}{2}(H_{22}-iH_{23}-iH_{23}^{*}-H_{33}) \end{pmatrix}\begin{pmatrix} \frac{1}{\sqrt{2}}(H_{12}^{*}+iH_{13}^{*}) & \frac{1}{2}(H_{22}+iH_{23}+iH_{23}^{*}-H_{33}) \end{pmatrix}}{E-\frac{1}{2}(H_{22}-iH_{23}+iH_{23}^{*}+H_{33})+\lambda}, \quad (11)$$

Substituting the matrix elements in Supplementary Equation. (4) into Supplementary Equation (11) and expanding k to the leading order around the Γ point, we obtain an effective Hamiltonian $H_{\text{eff}} = d_0 I + \mathbf{d}\cdot\boldsymbol{\sigma}$, where I is the identity matrix, $\boldsymbol{\sigma}$ is the Pauli matrices and

$$\begin{aligned} d_0 &= \frac{1}{2}[\varepsilon_s+\varepsilon_p+6t_{ss\sigma}+3(t_{pp\pi}+t_{pp\sigma})+\lambda] \\ d_x &= -\frac{3}{\sqrt{2}}t_{sp\sigma}k_y \\ d_y &= -\frac{3}{\sqrt{2}}t_{sp\sigma}k_x \\ d_z &= \frac{1}{2}[\varepsilon_s-\varepsilon_p+6t_{ss\sigma}-3(t_{pp\pi}+t_{pp\sigma})-\lambda] \end{aligned}, \quad (12)$$

Similarly, starting from Supplementary Equation (5), if we eliminate the bottom (s) orbital for the second type band ($E_s < E_p$), the effective two-band Hamiltonian becomes

$$H_{\text{eff}} = \begin{pmatrix} \frac{1}{2}(H_{22} + iH_{23} - iH_{23}^* + H_{33}) + \lambda & \frac{1}{2}(H_{22} - iH_{23} - iH_{23}^* - H_{33}) \\ \frac{1}{2}(H_{22} + iH_{23} + iH_{23}^* - H_{33}) & \frac{1}{2}(H_{22} - iH_{23} + iH_{23}^* + H_{33}) - \lambda \end{pmatrix} + \frac{\begin{pmatrix} \frac{1}{\sqrt{2}}(H_{12}^* - iH_{13}^*) \\ \frac{1}{\sqrt{2}}(H_{12}^* + iH_{13}^*) \end{pmatrix} \begin{pmatrix} \frac{1}{\sqrt{2}}(H_{12} + iH_{13}) & \frac{1}{\sqrt{2}}(H_{12} - iH_{13}) \end{pmatrix}}{E - H_{11}}, \quad (13)$$

Substituting the matrix elements in Supplementary Equation (4) into Supplementary Equation (13) and expanding k to the leading order around the Γ point, we can obtain an effective Hamiltonian $H_{\text{eff}} = d_0 I + \mathbf{d} \cdot \boldsymbol{\sigma}$, where

$$\begin{aligned} d_0 &= \varepsilon_p + 3(t_{pp\pi} + t_{pp\sigma}) - [\frac{3}{4}(t_{pp\pi} + t_{pp\sigma}) - \frac{9t_{sp\sigma}^2 / 2}{E - (\varepsilon_s + 6t_{ss\sigma})}](k_x^2 + k_y^2) \\ d_x &= [\frac{3}{8}(t_{pp\pi} - t_{pp\sigma}) + \frac{9t_{sp\sigma}^2 / 2}{E - (\varepsilon_s + 6t_{ss\sigma})}](k_x^2 - k_y^2) \\ d_y &= [\frac{3}{4}(t_{pp\pi} - t_{pp\sigma}) + \frac{9t_{sp\sigma}^2}{E - (\varepsilon_s + 6t_{ss\sigma})}]k_x k_y \\ d_z &= \lambda \end{aligned}, \quad (14)$$

Using the $\mathbf{d}$ vector, the Chern number can be further defined as

$$C = \frac{1}{4\pi} \int dk_x \int dk_y \hat{\mathbf{d}} \cdot (\frac{\partial \hat{\mathbf{d}}}{\partial k_x} \times \frac{\partial \hat{\mathbf{d}}}{\partial k_y}). \quad (15)$$

where $\hat{\mathbf{d}} = \mathbf{d} / |\mathbf{d}|$.
